# MicroRNA-194 reciprocally stimulates osteogenesis and inhibits adipogenesis via regulating COUP-TFII expression

**DOI:** 10.1038/cddis.2014.485

**Published:** 2014-11-20

**Authors:** B-C Jeong, I-H Kang, Y-C Hwang, S-H Kim, J-T Koh

**Affiliations:** 1 Research Center for Biomineralization Disorders, and Dental Science Research Institute, School of Dentistry, Chonnam National University, Gwangju, Korea; 2 Department of Pharmacology and Dental Therapeutics, School of Dentistry, Chonnam National University, Gwangju, Korea; 3 Department of Conservative Dentistry, School of Dentistry, Chonnam National University, Gwangju, Korea; 4 Department of Oral Anatomy, School of Dentistry, Chonnam National University, Gwangju, Korea

## Abstract

Osteoblasts and adipocytes are differentiated from common mesenchymal stem cells (MSCs) in processes which are tightly controlled by various growth factors, signaling molecules, transcriptional factors and microRNAs. Recently, chicken ovalbumin upstream promoter-transcription factor II (COUP-TFII) was identified as a critical regulator of MSC fate. In the present study, we aimed to identify some microRNAs (miR), which target COUP-TFII, and to determine the effects on MSCs fate. During osteoblastic or adipocytic differentiation from MSCs lineage cells, miR-194 expression was found to be reversal. In the cultures of mesenchymal C3H10T1/2 and primary bone marrow stromal cells, osteogenic stimuli increased miR-194 expression with accompanying decreases in COUP-TFII expression, whereas adipogenic stimuli reduced miR-194 expression with accompanying increases in COUP-TFII expression. A luciferase assay with COUP-TFII 3′-untranslated region (UTR) reporter plasmid, including the miR-194 binding sequences, showed that the introduction of miR-194 reduced the luciferase activity. However, it did not affect the activity of mutated COUP-TFII 3′-UTR reporter. Enforced expression of miR-194 significantly enhanced osteoblast differentiation, but inhibited adipocyte differentiation by decreasing COUP-TFII mRNA and protein levels. In contrast, inhibition of the endogenous miR-194 reduced matrix mineralization in the MSCs cultures, promoting the formation of lipid droplets by rescuing COUP-TFII expression. Furthermore, overexpression of COUP-TFII reversed the effects of miR-194 on the cell fates. Taken together, our results showed that miR-194 acts as a critical regulator of COUP-TFII, and can determinate the fate of MSCs to differentiate into osteoblasts and adipocytes. This suggests that miR-194 and COUP-TFII may be good target molecules for controlling bone and metabolic diseases.

Mesenchymal stem cells (MSCs) are pluripotent progenitors with multilineage differentiation potential, capable of undergoing osteogenesis, adipogenesis and chondrogenesis.^1, 2^ General bone homeostasis relies on the inverse relation between osteogenesis and adipogenesis of MSCs. The imbalance between osteogenesis and adipogenesis is associated with pathologic conditions such as osteoporosis, which is accompanied by an increase in bone marrow adipocytes and a decrease in bone mass.^3, 4^ Bone and fat development are largely dependent on the key transcription factors runt-related transcription factor 2 (Runx2) and peroxisome proliferator-activated receptor gamma (PPAR*γ*), respectively.^5, 6^ Moreover, Runx2 and PPAR*γ* may also control the lineage commitment and differentiation of MSCs. Runx2 deficiency in chondrocytes causes enhanced adipogenic differentiation,^7^ while PPAR*γ* haploinsufficient mice show an increase in bone mass associated with a loss of adipose tissue volume.^8^ In addition, transcriptional modulator TAZ interacts with Runx2 to promote osteogenesis, whereas binding of TAZ to PPAR*γ* impairs adipogenesis.^9^

Chicken ovalbumin upstream promoter-transcription factor II (COUP-TFII; NR2F2), a member of the orphan nuclear receptor superfamily, modulates mesenchymal cell commitment and differentiation to decide whether cells take part in osteogenesis, adipogenesis and chondrogenesis.^10, 11^ COUP-TFII activates PPAR*γ* expression and simultaneously inhibits Runx2 activity, thus promoting the entry of mesenchymal progenitors to the adipocyte lineage, while impeding progression to alternative lineage pathways.^10^ Conversely, ablation of COUP-TFII was found to increase bone formation and osteoblast differentiation, as well as reduce fat tissue and insulin sensitivity.^10^ Haploinsufficiency of COUP-TFII also displayed impairment of white adipose tissue development and reduced body fat mass.^11^ We previously demonstrated that COUP-TFII inhibited bone morphogenetic protein 2 (BMP2)-mediated osteoblast differentiation in mesenchymal C3H10T1/2 cells through the suppression of Runx2 activity.^12^ In addition, we observed that BMP2 reduced the cellular levels of COUP-TFII protein with osteoblast differentiation. These findings indicated that COUP-TFII is a negative regulator of bone formation, and a positive regulator of adipogenesis.

MiRNAs are an abundant class of noncoding, small (about 20~25 nucleotides) RNAs, which have emerged as key regulators of gene expression through translational inhibition and mRNA stability, and have been implicated in the regulation of cell proliferation, determination of cell fate or differentiation and organ development.^13^ Recently, several miRNAs were also discovered to be involved in the balance of osteogenesis and adipogenesis. For example, the increased expression of miR-204 and miR-637 were found to suppress osteoblast differentiation and enhance adipocyte differentiation by directly targeting Runx2 or Osterix.^14, 15^ Conversely, overexpression of miR-30e stimulated adipogenesis while inhibiting osteogenesis, by directly targeting low-density lipoprotein receptor-related protein 6.^16^ These observations suggested that osteoblast and adipocyte differentiation from mesenchymal progenitor cells is tightly regulated by various miRNAs. However, the specific miRNAs for targeting COUP-TFII and subsequently controlling the differentiation of pluripotent mesenchymal cells have not yet been fully clarified.

In this study, we found that COUP-TFII has a critical role in determination of the fate of MSCs, and that miR-194 reciprocally regulates osteogenesis and adipogenesis by directly targeting the 3′-UTR of COUP-TFII mRNA.

## Results

### Expression of miR-194 was differentially regulated during osteoblast and adipocyte differentiation

**Figure 1:**
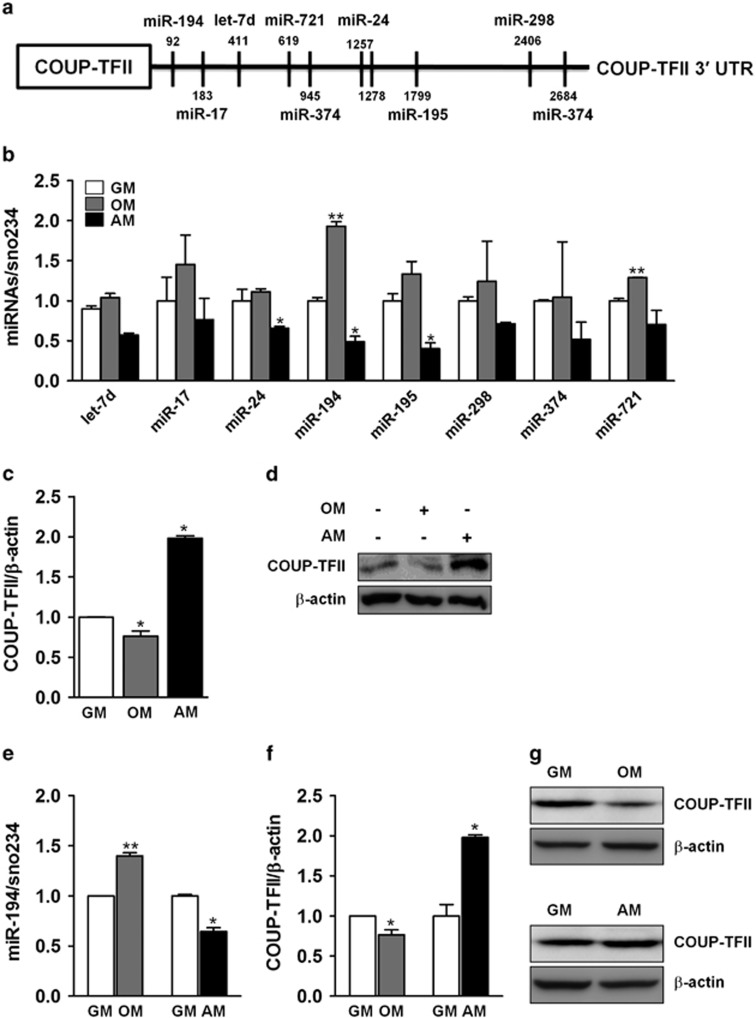
Expression of miR-194 was induced during osteoblast differentiation and reduced during adipocyte differentiation. (**a**) Schematic representation of the eight miRNAs predicted to target COUP-TFII-3′-UTR. (**b-d**) Mesenchymal C3H10T1/2 cells were cultured in osteogenic induction medium (OM) or adipogenic induction medium (AM) for 3 days. (**b**) Expression levels of COUP-TFII and miRNAs as determined by qRT-PCR (*n*=3). After normalizing against *β*-actin or sno234, and compared with that of growth medium (GM) control. Values represent mean±S.D. *, *P*<0.05. (**c**) Relative expression was calculated after normalization to *β*-actin or sno234 levels (*n*=3). Values represent mean±S.D. *, *P*<0.05 and **, *P*<0.01. (**d**) Western blotting analysis was performed for protein levels of COUP-TFII. *β*-actin was used as a loading control. (**e**, **f**) Primary BMSCs were cultured in OM or AM for 4 days. The expression levels of miR-194 and COUP-TFII were analyzed by qRT-PCR. Relative expression was calculated after normalization to *β*-actin or sno234 levels (*n*=3). Values represent mean±S.D. *, *P*<0.05 compared with growth medium cultures. (**g**) Western blotting analysis was performed for protein levels of COUP-TFII. *β*-actin was used as a loading control

COUP-TFII is a critical modulator controlling mesenchymal differentiation.^10^ To assess whether miRNAs could control COUP-TFII through binding to the 3′-UTR of COUP-TFII mRNA, miRNA target prediction programs (i.e., TargetScan (http://www.targetscan.org/), PicTar (http://pictar.mdc-berlin.de/) and RNA22 (https://cm.jefferson.edu/rna22v2/)) were applied to search for COUP-TFII-targeting miRNAs. A total of eight miRNAs were identified, including let-7d, miR-17, miR-24, miR-194, miR-195, miR-298, miR-374 and miR-721, which could potentially bind to the 3′-UTR of COUP-TFII mRNA (Figure 1a). To determine if the miRNAs expression is modulated with COUP-TFII expression during osteogenesis and adipogenesis, mesenchymal C3H10T1/2 cells were cultured in osteogenic medium (OM) or adipogenic medium (AM) for 3 days, after which qRT-PCR and Western blotting were performed. Results showed that the levels of COUP-TFII mRNA and protein expression were significantly decreased when cells were cultured with OM, while the expression was increased with AM treatment (Figures 1c and d). These findings were consistent with other reports.^10^ Consequently, the expression of miR−194, −195 and −721 were upregulated when cells underwent differentiation toward osteogenic lineage (Figure 1b). In contrast, let-7d, miR−24, −194, −195, −298 and miR-721 expression was robustly decreased by adipogenic stimuli (Figure 1b). Among those detected, miR-194 showed the greatest changes in both osteogenesis and adipogenesis, revealing a twofold induction by osteogenic and a 50% reduction by AM treatment, respectively. Also, similar results were obtained from primary mouse bone marrow stromal cells (BMSCs) (Figures 1e–g). Collectively, these results revealed that COUP-TFII and miR-194 expression was reversible during osteogenesis and adipogenesis of MSCs, suggesting that miR-194 may be a regulator of COUP-TFII in these cells.

### MiR-194 acts as an attenuator of COUP-TFII expression

**Figure 2:**
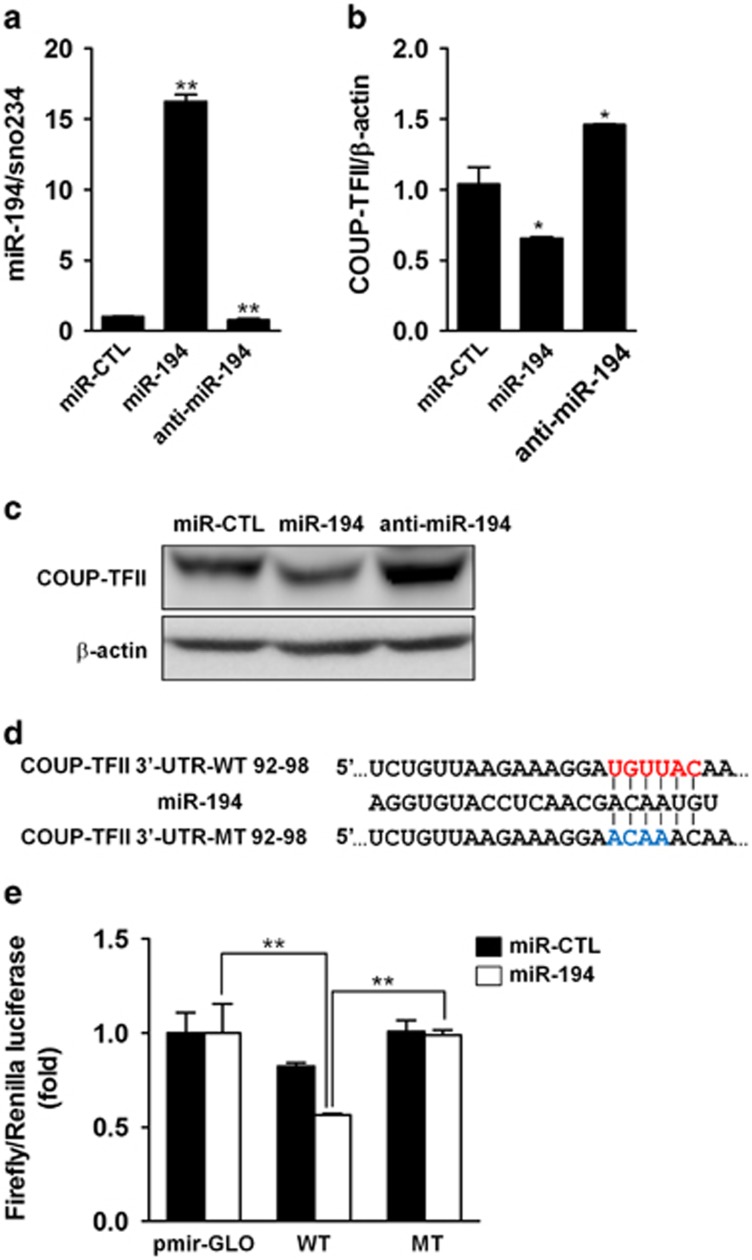
MiR-194 directly targets COUP-TFII mRNA. (**a, b**) C3H10T1/2 cells were transfected with miR-194 precursors or anti-miR-194. Expression levels of miR-194 and COUP-TFII mRNA were examined using qRT-PCR (*n*=3). Values represent mean±S.D. *, *P*<0.05 and **, *P*<0.01 compared with miR-CTL. (**c**) C3H10T1/2 cells were transfected with miR-194 precursors or anti-miR-194, and Western blotting analysis was performed. The data were obtained from three independent experiments. (**d**) Computational analysis of one putative complementary sequence for miR-194 in the 3′-UTR fragment of COUP-TFII. The wild type (WT) or mutant type (MT) construct was inserted into pmirGLO reporter vector. (**e**) The pmirGLO, pmirGLO COUP-TFII-3′-UTR-WT (WT) and pmirGLO COUP-TFII-3′-UTR-MT (MT) vectors were co-transfected with miR-CTL and miR-194 or anti-miR-194, respectively, into C3H10T1/2 cells. Luciferase activities were measured from the cell lysates after 24 h. Relative renilla luciferase activity was normalized to that of firefly luciferase. The values were normalized by the miR-CTL-treated group (*n*=3). **, *P*<0.01. All experiments were independently repeated at least three times

The effects of miR-194 on COUP-TFII expression were further examined by gain- and loss- of-function experiments in C3H10T1/2 cells. Introduction of miR-194 or anti-miR-194 was confirmed by qRT-PCR (Figure 2a). The expression of COUP-TFII mRNA was reduced by enhanced expression of miR-194, whereas it was increased by inhibition of miR-194 (Figure 2b). Furthermore, introduction of miR-194 led to a decrease in COUP-TFII protein level, whereas suppression of endogenous miR-194 increased in C3H10T1/2 cells (Figure 2c), suggesting that miR-194 targeted the COUP-TFII mRNA to suppress its protein expression.

To further determine whether miR-194 directly targets the 3′-UTR of COUP-TFII mRNA, sequences of wild type COUP-TFII-3′-UTR luciferase reporter plasmid (WT), containing the putative miR-194 binding site and mutant COUP-TFII-3′-UTR (MT), with a 4 bp mutation in the seed region, were synthesized (Figure 2d). The WT- or MT-COUP-TFII 3′-UTR luciferase reporter plasmids were co-transfected with miR-194, control miRNA or anti-miR-194 into C3H10T1/2 cells, after which luciferase activity assays were performed. The co-transfection of miR-194 with a WT reporter resulted in a highly significant decrease in luciferase activity compared with the control-transfected group (Figure 2e). When the miR-194 was co-transfected with the empty vector or MT reporter, no decrease in luciferase activity was observed, confirming that the predicted site is a direct target of miR-194. Collectively, these results revealed that miR-194 negatively regulate the expression of COUP-TFII by directly binding to the COUP-TFII-3′-UTR.

### MiR-194 promotes osteoblast differentiation

**Figure 3:**
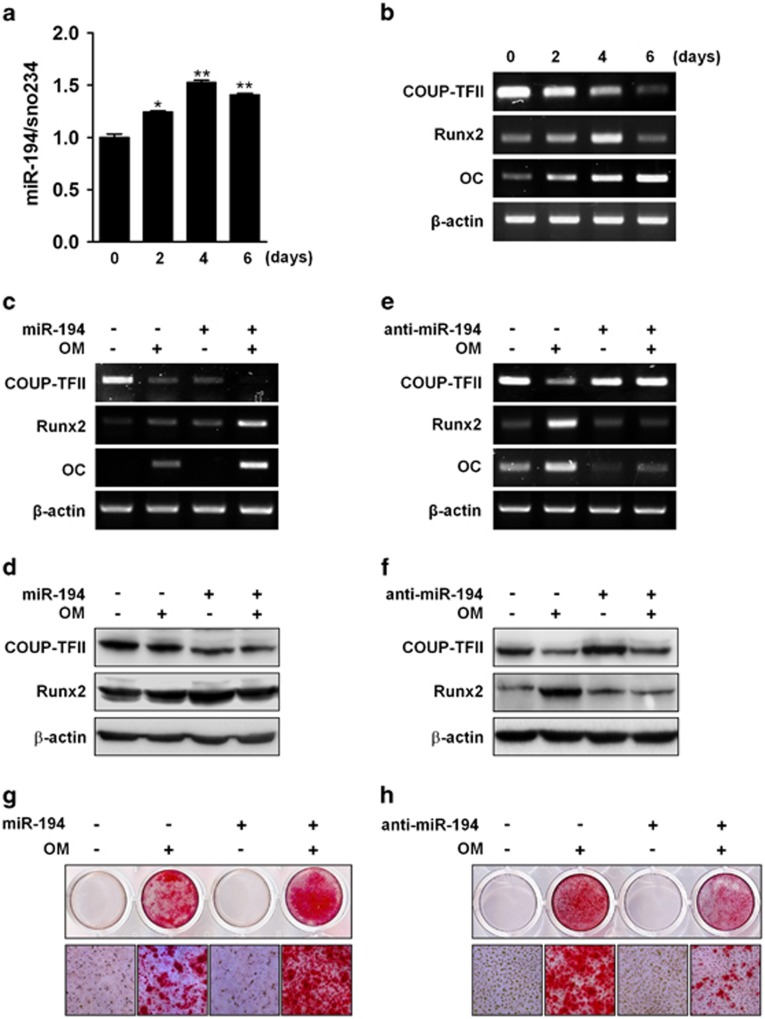
MiR-194 promotes osteoblasts differentiation. (**a**, **b**) Primary osteoblasts were cultured in OM for the indicated time period. Total RNA was isolated, and the expression levels of miR-194 and osteogenic markers were evaluated by qRT-PCR or RT-PCR, respectively (*n*=3). *, *P*<0.05 and **, *P*<0.01 compared with day 0 group. (**c**–**f**) C3H10T1/2 cells were transfected with miR-194 precursors or anti-miR-194 for 24 h, and then treated with OM for an additional 4-day period before performing RT-PCR (**c**, **e**) or Western blot analysis (**d**, **f**) with the indicated primers or antibodies. All data presented were independently repeated three times. (**g**, **h**) C3H10T1/2 cells were transfected with miR-194 precursor or anti-miR-194 for 24 h, and then treated with OM for an additional 7-day period before alizarin red staining was performed. A representative image of three independent experiments was shown

Because miR-194 was found to have an inhibitory effect on COUP-TFII expression, we investigated whether miR-194 controls osteoblast differentiation. The expression levels of miR-194 were first examined at different time-points of osteogenic differentiation in primary osteoblasts. The miR-194 expression increased following osteogenic differentiation, reaching a high on the 4th day (Figure 3a). At the time-points, expression of osteogenic markers such as Runx2 and osteocalcin (OC) were gradually increased with the deceased expression of COUP-TFII (Figure 3b). To further verify whether miR-194 could regulate osteogenesis through the regulation of COUP-TFII, the expression of miR-194 was altered by transfecting C3H10T1/2 cells with a specific synthetic miRNA precursor or inhibitor (Supplementary Figure S1). Transfection of miR-194 precursor elicited to increasing Runx2 and OC mRNA expressions, whereas inhibition of miR-194 with anti-miR-194 suppressed the expression in the presence or absence of OM (Figures 3c and e). Furthermore, introduction of miR-194 decreased COUP-TFII protein levels, while inhibition of miR-194 caused them to increase. The introduction of miR-194 enhanced Runx2 protein levels, and transfection with anti-miR-194 reduced Runx2 protein levels (Figures 3d and f). In addition, overexpression of miR-194 promoted matrix mineralization, whereas knockdown reduced mineralization (Figures 3g and h), as evidenced by alizarin red staining. Collectively, these results suggested miR-194 as a positive regulator of osteoblast differentiation, possibly by the downregulation of COUP-TFII expression.

### MiR-194 inhibits adipocyte differentiation

**Figure 4:**
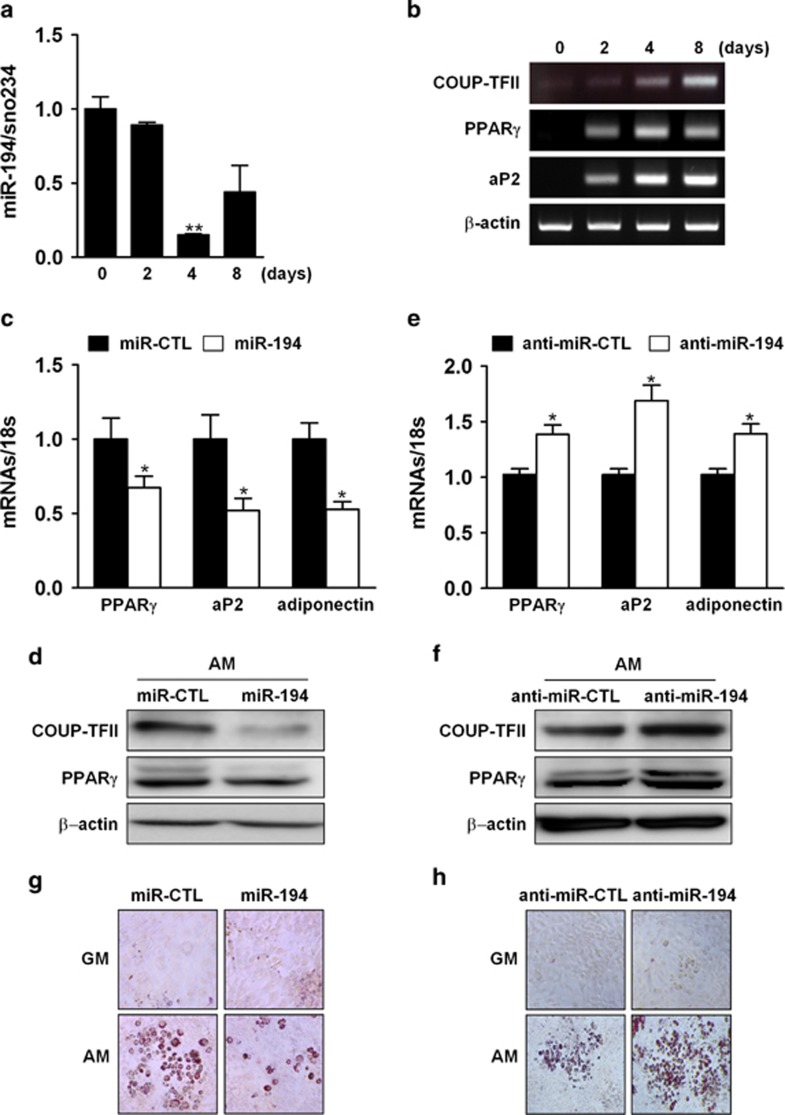
MiR-194 suppresses adipocytes differentiation. (**a**, **b**) 3T3-L1 cells were cultured in AM for the indicated time period, after which total RNA was isolated and expression levels of miR-194 and adipogenic markers were evaluated by qRT-PCR or RT-PCR, respectively (*n*=3). **, *P* < 0.01 compared with day 0 group. (**c**, **e**) 3T3-L1 cells were transfected with miR-194 precursors or anti-miR-194 for 24 h, and then cultured in AM for an additional 4-day period before performing RT-PCR with indicated primers, respectively (*n*=3). *, *P*<0.05 compared with the miR-CTL group. (**d**, **f**) Western blot analysis with indicated antibodies. (**g**, **h**) 3T3-L1 cells were transfected with miR-194 precursors or anti-miR-194 for 24 h and then treated with AM for an additional 7-day period before performing Oil Red O staining. A representative image of three independent experiments was shown

The effects of miR-194 on adipocyte differentiation were also investigated in 3T3-L1 cells. During the adipocyte differentiation, expression of miR-194 decreased and reached a minimum on day 4 (Figure 4a). On the other hand, COUP-TFII expression was gradually increased up to 8 day with the increased expression of adipogenic marker aP2 and PPAR*γ* (Figure 4b). In gain- or loss-of-function experiments, introduction of miR-194 elicited to decreasing expression of PPAR*γ*, aP2 and adiponectin mRNA, whereas the inhibition of miR-194 increased them (Figures 4c and e,Supplementary Figure S2). Western blot analysis also revealed that miR-194 reduced PPAR*γ* and COUP-TFII protein levels, and that anti-miR-194 increased them (Figures 4d and f). Consistently, overexpression of miR-194 reduced lipid droplet formation in 3T3-L1, and reversely anti-miR-194 enhanced it (Figures 4g and h), as evidenced by the Oil Red O staining. These results suggested that miR-194 negatively regulates adipocyte differentiation through suppressing COUP-TFII expression.

### MiR-194 regulates lineage specification of mesenchymal cells via COUP-TFII

**Figure 5:**
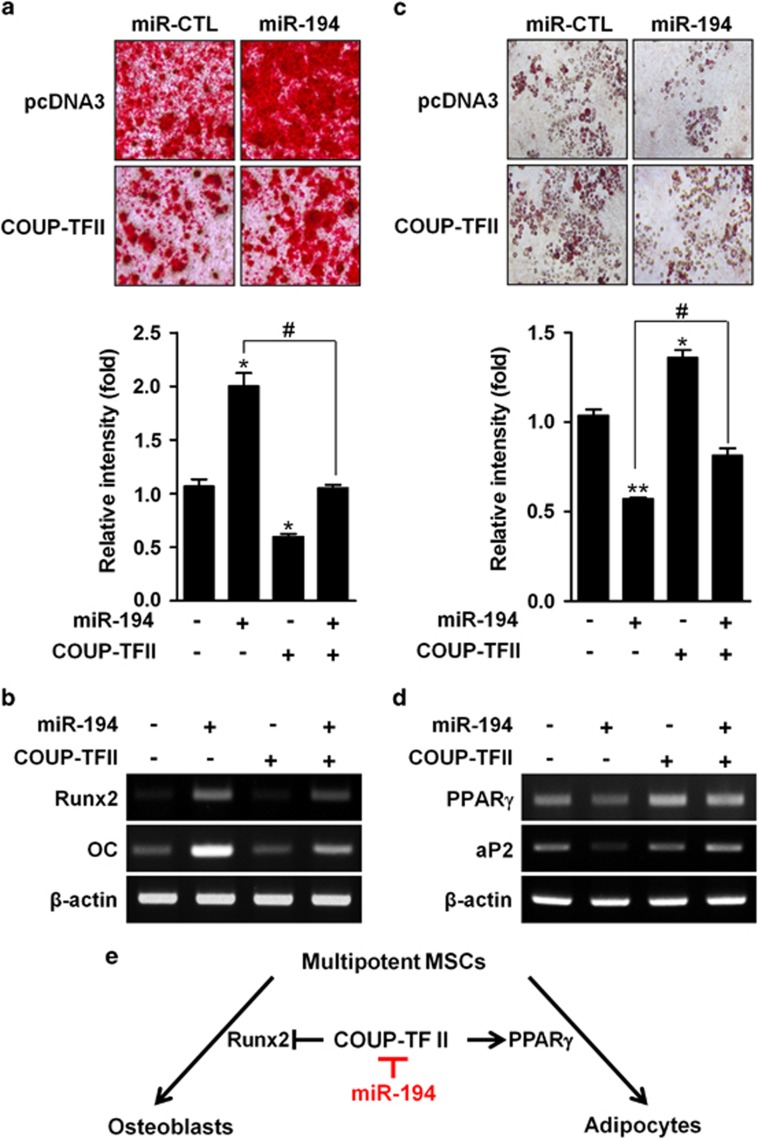
MiR-194 can regulate osteogenesis and adipogenesis from mesenchymal cells. (**a**) C3H10T1/2 cells were co-transfected with miR-194 (10 nM) or miR-CTL (10 nM) and COUP-TFII expression vector without 3′-UTR (100 ng) or control pcDNA3 vector (100 ng), cultured in OM for 6 days, and then subjected to alizarin red staining. The levels of calcium deposition was quantified by using image J software (*n*=3). *, *P* < 0.05 compared with the control group. ^#^, *P* < 0.05 compared with the indicated group. (**b**) Expression levels of osteogenic markers were determined by RT-PCR after culturing in OM for 4 days. (**c**) 3T3-L1 cells were co-transfected with miR-194 (40 nM) or miR-con (40 nM) and the COUP-TFII expression vector (100 ng) or control pcDNA3 vector (100 ng), cultured in AM for 6 days and then subjected to Oil Red O staining. The levels of oil drop was quantified by using image J software (National Institutes of Health, Bethesda, MD, USA) (*n*=3). *, *P* < 0.05 and **, *P* < 0.01 compared with the control group. ^#^, *P* < 0.05 compared with the indicated group. (**d**) Expression levels of adipogenic markers were determined by RT-PCR. (**e**) Schematic diagram of miR-194-mediated regulation of osteoblast and adipocyte differentiation from MSCs

All the above results suggest that COUP-TFII may be a direct target of miR-194. To further confirm whether COUP-TFII directly mediates the miR-194 control of mesenchymal cell differentiation, we examined the effects of COUP-TFII overexpression on the miR-194 control of osteogenesis and adipogenesis. COUP-TFII expression vector, not including 3′-UTR, was co-transfected with miR-194 or control miRNA into mesenchymal C3H10T1/2 cells, after which qRT-PCR was performed (Supplementary Figure S3). The supplementation of COUP-TFII inhibited the promoting effect of miR-194 on mineralization with attenuating miR-194-induced Runx2 and OC expression (Figures 5a and b). In addition, the supplementation relived the miR-194 inhibition of lipid droplet formation with the increased expression of aP2 and PPAR*γ* in mesenchymal lineage cells (Figures 5c and d). These results suggest that miR-194 is critical for maintaining the balance of adipocyte and osteoblast differentiation from MSC through the regulation of COUP-TFII (Figure 5e).

## Discussion

Osteoblasts and adipocytes arise from a common mesenchymal progenitor, and lineage allocation into osteoblasts and adipocytes is reciprocally exclusive.^17^ Decreased bone formation which occurs with advanced age is usually accompanied by an accumulation of bone marrow adiposity.^18, 19^ Thus, the balance between osteogenesis and adipogenesis may be of great importance to prevent and treat metabolic disorders that involve bone loss and lipid accumulation.^20^ The activation of multiple transcription factors has been identified to be associated with determination of the fate of mesenchymal cells.^21, 22^ These findings imply that targeting transcription factors may be one of strategy for controlling the commitment and differentiation of MSCs to a certain lineage.

COUP-TFII is one of critical transcription factors to determine the lineage specification of mesenchymal progenitors.^10^ COUP-TFII activates PPAR*γ* expression while inhibiting Runx2 activity, thus promoting the entry of progenitor cells into the adipocyte lineage, while impeding the access of MSCs to alternative lineages.^12, 23^ Recently, some miRNAs, especially targeting transcription factors, have emerged as one of the fate determinants of MSC. For example, miR-204 and miR-637 can control the MSC commitment through regulating Runx2 and OSX expression, which are major transcription factors for bone formation.^14, 15^

MiR-194 has been characterized as a regulatory mediator in liver fibrogenesis and metabolism of hepatic cholesterol and bile acid.^24^ In addition, miR-194 inhibits chondrogenic differentiation of human adipose-derived stem cells by targeting Sox5,^25^ and suppresses osteosarcoma cell proliferation and metastasis *in vitro* and *in vivo* by targeting CDH2 and IGF1R.^26^ These findings suggest that miR-194 might be a putative target molecule for regulating metabolic and bone disease.

In the present study, we identified a novel function of miR-194 concerning the regulation of COUP-TFII-mediated osteogenesis and adipogenesis. The miR-194 is preferentially expressed in bone compared with other tissues in mice (Supplementary Figure S4), suggesting that it may also have a regulatory role in bone development and homeostasis. Furthermore, miR-194 expression was upregulated during the differentiation of MSCs toward an osteogenic lineage, while downregulated during adipogenesis. Overexpression of miR-194 also promoted osteoblast differentiation of primary osteoblasts and mesenchymal lineage cells with and inhibited adipocyte differentiation of 3T3-L1. In contrast, the knockdown of miR-194 produced the opposite effects. The miR-194 control of osteoblast and adipocyte differentiation was related to the expression of COUP-TFII, Runx2 and PPAR*γ* (Figures 3 and 4). These results suggest that miR-194 might be a critical regulator in maintaining the balance between osteogenesis and adipogenesis.

MiRNAs control cellular activity by directly targeting the 3′-UTR of target mRNA with base-pair complementarity.^13^ Bioinformatic analyses informed us that 3′-UTR of COUP-TFII mRNA has complementary sequence to miR-194, and luciferase assays provided evidence that miR-194 targets the COUP-TFII mRNA in cellular level (Figure 2). In addition, our results consistently showed that the levels of COUP-TFII mRNA and protein were regulated by miR-194 in osteogenesis and adipogenesis processes. The miR-194 reduction of COUP-TFII expression induced osteogenesis and inhibited adipogenesis (Figures 3 and 4). In addition, inhibition of COUP-TFII by siRNA also produced the same phenomenon (Supplementary Figure S5), and COUP-TFII overexpression inhibited the effects of miR-194 on osteogenesis and adipogenesis. These results strongly support our previous reports that COUP-TFII is a negative regulator of osteoblast differentiation,^12^ and also indicate that COUP-TFII may have a stimulatory role in adipogenesis.

Runx2 is involved in the lineage commitment and differentiation between osteoblasts and adipocytes, and also recently identified as a target molecule of COUP-TFII.^12^ In the study, we also found that miR-194 overexpression was accompanied by decreased levels of COUP-TFII expression, whereas knockdown of miR-194 increased the COUP-TFII protein level (Figure 3). These findings provided evidence that COUP-TFII may regulate osteogenic differentiation by directly interacting with the Runx2 protein to suppress osteogenic-related genes, which could be considered as one of the mechanisms underlying miR-194-mediated regulation of osteogenic differentiation.

PPAR*γ* is also considered as a crucial regulator between adipogenesis and osteogenesis. PPAR*γ* repressed Runx2-mediated OC transcription,^27^ and its insufficiency enhances bone mass through osteoblast formation of bone marrow progenitors.^8^^28^ Adipocytes also directly modulate osteoblast function through paracrine effects of secretory adipokines, such as adiponectin and leptin, which are regulated by PPAR*γ*.^29, 30^ Our study also revealed that miR-194 controlled adipogenesis as well as osteogenesis with alteration of COUP-TFII and PPAR*γ* expression. Indeed, inhibition of miR-194 elicited to decreasing Runx2 expression and increasing PPAR*γ* expression in mesenchymal cells under adipogenic conditions (Supplementary Figure S6), suggesting inhibition of miR-194 may commit MSC into adipocytes over osteoblasts.

In conclusion, we demonstrated as a novel regulatory mechanism of mesenchymal progenitor cells that miR-194 stimulated osteogenesis and inhibited adipogenesis from MSC via regulating COUP-TFII expression (Figure 5e). Therefore, miR-194 may prove to be a promising therapeutic target for treatment of metabolic and bone disease, including osteoporosis or obesity.

## Materials and Methods

### Reagents and microRNA precursor

The recombinant human BMP2 peptide was obtained from Daewoong Pharmaceutical (Seoul, Korea). Oligonucleotides (pri-miR negative control (miR-CTL), anti-miR negative control (anti-miR-CTL), pri-miR-194 precursor (miR-194), and miR-194 inhibitor (anti-miR-194)) and mirVana miRNA isolation kits were purchased from Ambion (Austin, TX, USA). Dual-Luciferase miRNA target expression vector (pmirGLO) was obtained from Promega (Madison, WI, USA).

### Cell culture

Bone marrow cells were isolated from the tibias and femurs of 8-week-old mice. BMSCs were cultured in *α*-minimal essential medium (*α*-MEM; GIBCO-BRL, Grand Island, NY, USA) with 15% fetal bovine serum (FBS, Invitrogen, Carlsbad, CA, USA), supplemented with 100 U/ml of penicillin (Invitrogen) and 100 *μ*g/ml of streptomycin (Invitrogen), and then grown in *α*-MEM with 10% FBS for 2–3 days. Primary calvarial cells were prepared from newborn mice by sequential collagenase digestion, as described in the literature.^31^ Calvarial cells and mouse preosteoblast MC3T3-E1 cells were cultured in *α*-MEM supplemented with 10% FBS, 100 U/ml of penicillin (Invitrogen) and 100 *μ*g/ml of streptomycin (Invitrogen). Murine mesenchymal C3H10T1/2 cells and preadipocyte 3T3-L1 cells were cultured in Dulbecco's modified Eagle's medium (DMEM; GIBCO-BRL).

### Osteoblast and adipocyte differentiation

To induce osteoblast differentiation, cells were cultured with OM containing 10% FBS, 50 *μ*g/ml ascorbic acid and 5 mM *β*-glycerophosphate, in the presence or absence of 200 ng/ml of BMP2. The induction media was changed every 3 days. To induce adipocyte differentiation, cells were allowed to become confluent for 1 day, and then cultured with AM containing 0.5 mM 3-isobutyl-1-methylxanthine, 1 *μ*M dexamethasone and 1 *μ*g/ml insulin. After 2 days, the medium was replaced with fresh complete medium containing insulin, and the cells were incubated for an additional 2-day period. Thereafter, the cells were maintained in 10% FBS-DMEM with media changes every other day until they became fully differentiated.

### DNA constructions

COUP-TFII expression vector without 3′-UTR, shCOUP-TFII and shLuc control vector was previously described.^12^ By *in silico* analysis, COUP-TFII-3′-UTR was found to have a putative miR-194 binding site (UGUUACA; 92–98 bp far from stop codon; TargetScan). For functional analysis of miR-194, pmirGLO-COUP-TFII-3′-UTR-Luc constructs containing a partial fragment of COUP-TFII-3′-UTR and luciferase reporter genes were made. Amplification of wild-type and mutant plasmids was carried out under the same conditions. Cloning was carried out using specific forward and reverse primers (Supplementary Table 1). The fragments of 3′-UTR were sub-cloned between the *Sac*I and *Xho*I restriction sites of the pmirGLO vector (Promega). The correct orientation and nucleotide sequence of the 3′-UTR fragments in the plasmid constructs were further confirmed by sequencing.

### Transient transfection and luciferase assays

Cells were transfected transiently with the indicated plasmids, 20 nM miR-194 (or 20 nM miR-CTL) and 40 nM anti-miR-194 (or 40 nM anti-miR-CTL) using Lipofectamin RNAiMAX (Invitrogen). To measure promoter activity, cells were harvested 24–48 h after transfection, and the activity was measured with the luciferase reporter assay system (Promega). To confirm interaction between miR-194 and the COUP-TFII-3′-UTR region, cells were transfected with 100 ng of pmirGLO wild type (or 100 ng mutant type) or empty pmirGLO vector (100 ng) in the presence of 10 nM miR-194 precursor (or 10 nM miR-CTL) using Lipofectamine 2000 (Invitrogen, Carlsbad, CA, USA). Thereafter, firefly and renilla luciferase activities were determined using the Dual-Glo luciferase assay system (Promega).

### RT-PCR and qRT-PCR

Total RNA or microRNAs (miRs) were isolated from the cultures using the TRIzol reagent (Invitrogen) or the mirVana miRNA isolation kit (Ambion). For quantitation of gene transcription, cDNA was generated with the Maxime RT premix kit (iNtRon, Sungnam, Korea), and then amplified on the StepOnePlus real-time PCR system (ABI, Abilene, TX, USA) using the QuantiTect SYBR PCR kit (Qiagen, Valencia, CA, USA) with specific primers (Supplementary Table 2). To quantify gene expression of adipogenc markers, qRT-PCR was performed using TaqMan probes with TaqMan Universal PCR Master Mix (Applied Biosystems, Foster city, CA, USA) in the StepOnePlus real-time PCR System (Applied Biosystems). Unlabeled specific primers and the TaqMan probes were purchased from Applied Biosystems for detecting the aP2 gene (Assay ID: Mm00445878_m1), adiponectin gene (Assay ID: Mm00456425_m1) and PPAR*γ* gene (Assay ID: Mm01184322_m1). All quantitation was normalized to an endogenous control 18S gene (Assay ID: Hs99999901_s1). MiRNAs were evaluated using the NCode VILO miRNA cDNA synthesis kit (Invitrogen) and the Express SYBR GreenER miRNA qRT-PCR kit with specific primers (Supplementary Table 3). The relative level of miRNA was quantified using the 2^−ΔΔCt^ method with sno234 RNA as an endogenous control.

### Western blot analysis

Total cell extracts were harvested in lysis buffer (Cell Signaling Technology, Danvers, MA, USA) and centrifuged at 12 000 × *g* for 15 min at 4 °C. Quantification of total protein was performed using the BCA protein assay reagent (Bio-Rad Laboratories, Hercules, CA, USA). Proteins were resolved by 10% SDS-PAGE and transferred to a PVDF membrane. After blocking in Tris-buffered saline with 5% milk and 0.1% Tween-20, the membrane was incubated with primary antibodies for COUP-TFII (1 : 1000, Abcam, Cambridge, MA, USA), Runx2 (1 : 1000, Santa Cruz Biotechnology, Santa Cruz, CA, USA), PPAR*γ* (1 : 1000, Santa Cruz Biotechnology) and *β*-actin (1 : 2000, Cell Signaling Technology). Signals were visualized using an enhanced chemiluminescence reagent (Santa Cruz Biotechnology) in a LAS-4000 luminoimage analyzer system (Fujifilm, Tokyo, Japan).

### Alizarin red staining

Cells were fixed with 70% ethanol for 1 h, rinsed with cold distilled water, and then treated with 40 mM AR-S solution at pH 4.2 for 10 min. After washing with phosphate-buffered saline for 15 min, the stained cultures were photographed. To quantify calcium deposition, cells were washed with distilled water, the dye was eluted with 10% cetylpyridinium chloride, and the absorbance was measured at 570 nM with a microplate reader (Bio-Rad).

### Oil Red O staining

Cells were transfected with miR-194 precursors or anti-miR-302a using Lipofectamin RNAiMAX (Invitrogen), and then cultured in AM for 8 days. After washing with PBS, the cells were fixed with 4% formalin in PBS for 30 min, and then reacted with 60% saturated Oil Red O dye for 20 min. The Oil Red O-stained lipid droplets were photographed by LSM microscopy (Carl Zeiss, Oberkochen, Germany). To quantify the degree of lipid droplet formation, the stained cells were incubated with isopropanol to extract the dye from the lipid droplets, and the absorbance of reaction solution was then measured at 510 nM with a microplate reader (Bio-Rad).

### Statistical analysis

All experiments were performed in triplicate on the same sample, and independently repeated at least three times. Results were expressed as mean±S.D. Statistical analyses were performed using a Student's *t*-test or analysis of variance, followed by Duncan's multiple comparison tests. *P*-values<0.05 were considered to be statistically significant.

## Abbreviations

BMP2: bone morphogenetic protein 2
MiRNA: microRNA
MSC: mesenchymal stem cell
PPARγ: peroxisome proliferate-activated receptor gamma
Runx2: runt-related transcription factor 2
UTR: untranslated region
