# Supplementary material for: MicroRNA-194 reciprocally stimulates osteogenesis and inhibits adipogenesis via regulating COUP-TFII expression
Source: Cell Death Dis. 2014 Nov 20;5(11):e1532–. doi: 10.1038/cddis.2014.485 (PMC4260743; doi:10.1038/cddis.2014.485)
Supplement: Supplementary Figure Legends [file cddis2014485x1.docx]

**Supplementary figure legends**

**Supplementary Figure S1** Verification of miR-194 expression in transfected cells. C3H10T1/2 cells were transfected with miR-CTL (-) or anti-miR-CTL (-) and miR-194 (20 nM) or anti-miR-194 (40 nM) for 48 h. (**A** and **B**) Expression levels of miR-194 in the transfected cells were examined using qRT-PCR. The sno234 was used as an endogenous control (n=3). All values represent means ± SD. *, *p* < 0.05 and ***, *p* < 0.001.

**Supplementary Figure S2** Determination of miR-194 expression in transfected cells. 3T3-L1 cells were transfected with miR-CTL (-) or anti-miR-CTL (-) and miR-194 (20 nM) or anti-miR-194 (40 nM) for 48 h. (**A** and **B**) Expression levels of miR-194 in the transfected cells were examined using qRT-PCR. The sno234 was used as an inner control (n=3). All values are presented as means ± SD. *, *p* < 0.05.

**Supplementary Figure S3** Confirmation of miR-194 and COUP-TFII expression in transfected cells. Cells were co-transfected with miR-194 (10 nM or 40 nM) or miR-con (-; 10 nM or 40 nM) and the COUP-TFII expression vector not containing 3′-UTR (100 ng) or empty vector (-; 100 ng) for 4 days. (**A** and **B**) Expression levels of miR-194 and COUP-TFII were confirmed by qRT-PCR. Their relative expression was calculated after normalization to sno234 or β-actin levels, respectively (n=3). All values are presented as means ± SD. ***, *p* < 0.001 compared with control groups.

**Supplementary Figure S4** Expression profiles of miR-194 in various tissues. Total RNAs were isolated from each tissue collected from 8-week-old C57BL/6 mice. Equal amounts of total RNA were subjected to reverse transcription, and the levels of miR-194 expression were evaluated by qRT-PCR. The sno234 was used as an inner control (n=3).

**Supplementary Figure S5** The inhibitory effects of endogenous COUP-TFII are consistent with that of miR-194 overexpression on osteogenic and adipogeic differentiation. (**A**) The efficiency of shCOUP-TFII was confirmed by qRT-PCR in C3H10T1/2 cells. Values are presented as means ± SD. **, *p* < 0.01 compared with shLuc control group. (**B**) C3H10T1/2 cells were transfected with shCOUP-TFII or shLuc control, and then treated with OM or AM for an additional 6 or 8 days before ALP or Oil Red O staining, respectively. A representative image of the staining was shown.

**Supplementary Figure S6** The effects of anti-miR-194 on gene expression in adipogenic process of MSC differentiation. C3H10T1/2 cells transfected with anti-miR-194 or anti-miR-CTL were cultured in adipogenic medium for 4 days. (A) Expression levels of Runx2 and adipogenic markers were determined by qRT-PCR. 18S was used as an inner control (n=3). All values are presented as means ± SD. *, *p* < 0.05 compared with anti-miR-CTL groups. (**B**) Protein levels of Runx2 and PPARγ were determined by Western blot analysis.
