# Supplementary figures and images for: MicroRNA-194 reciprocally stimulates osteogenesis and inhibits adipogenesis via regulating COUP-TFII expression
Source: Cell Death Dis. 2014 Nov 20;5(11):e1532–. doi: 10.1038/cddis.2014.485 (PMC4260743; doi:10.1038/cddis.2014.485)

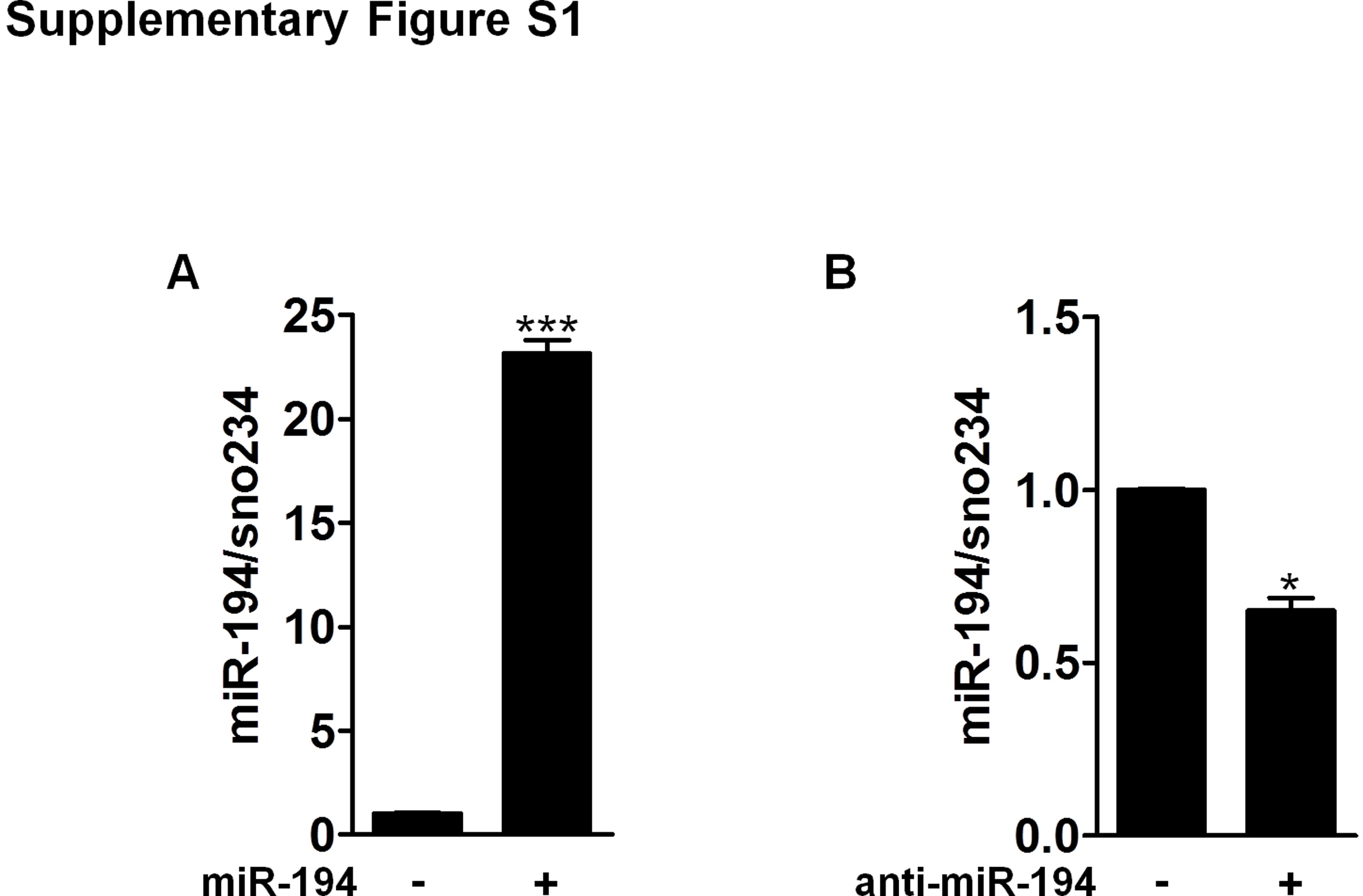

Supplement: Supplementary Figure S1 [file cddis2014485x2.tif]

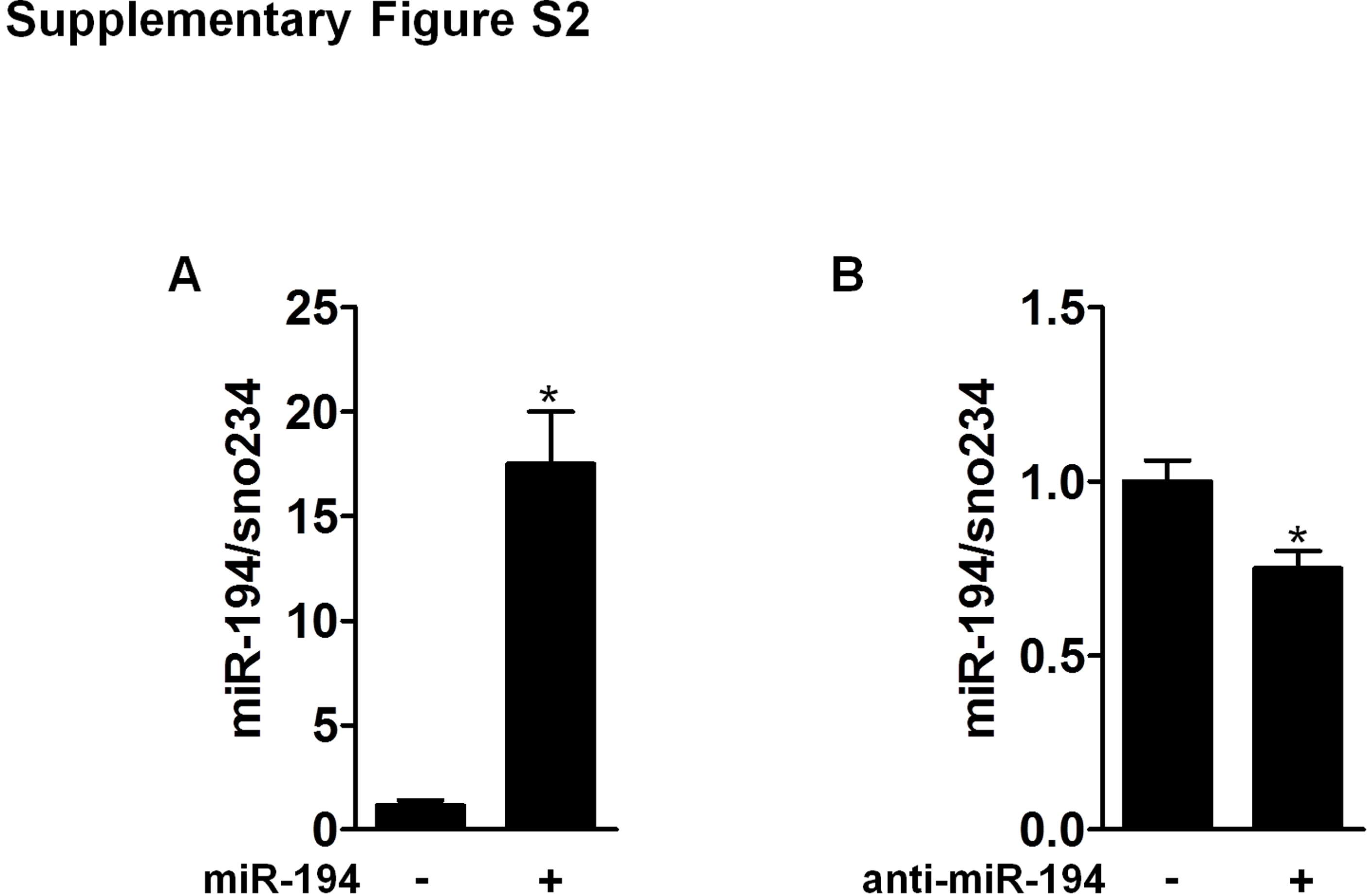

Supplement: Supplementary Figure S2 [file cddis2014485x3.tif]

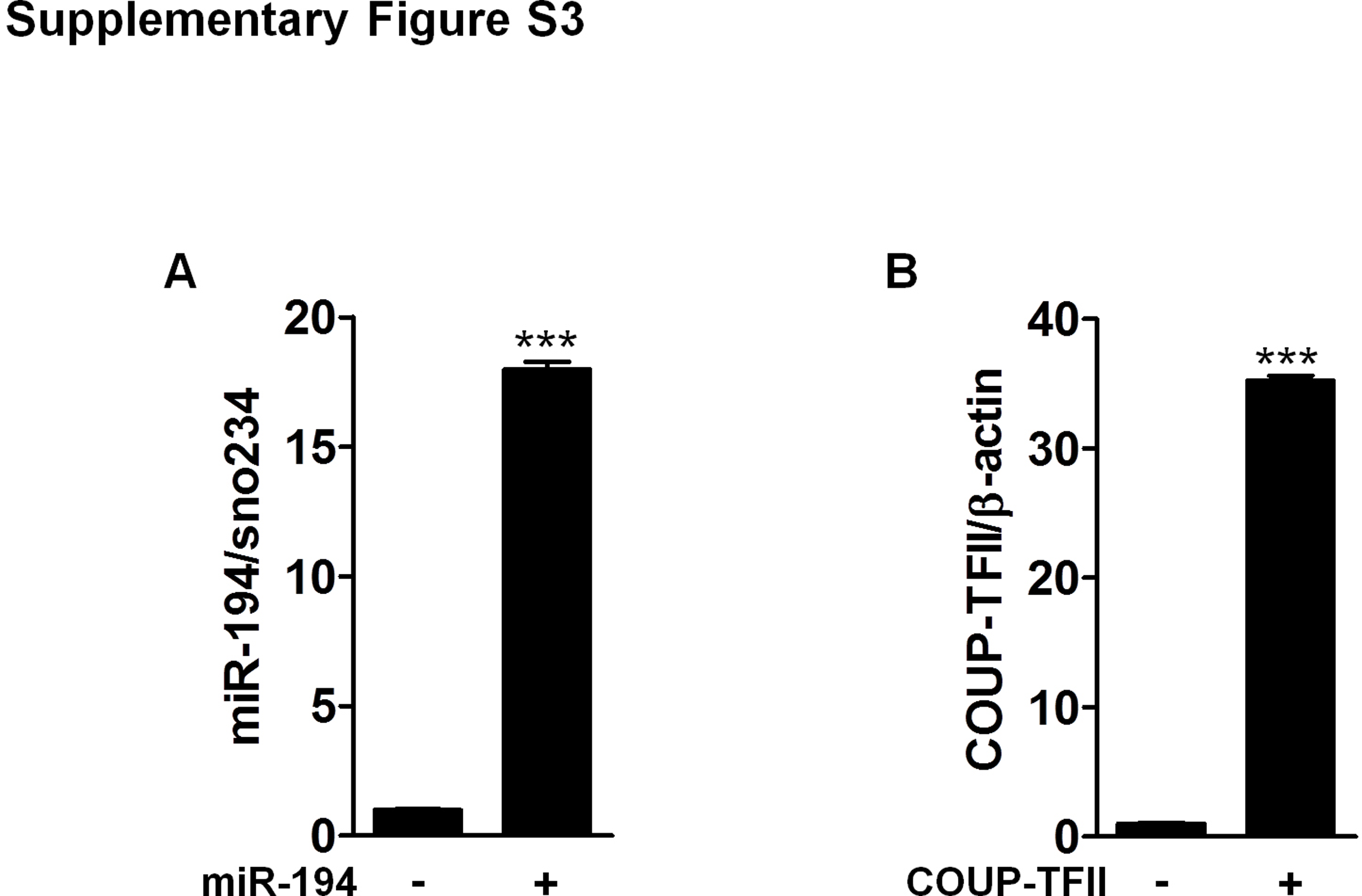

Supplement: Supplementary Figure S3 [file cddis2014485x4.tif]

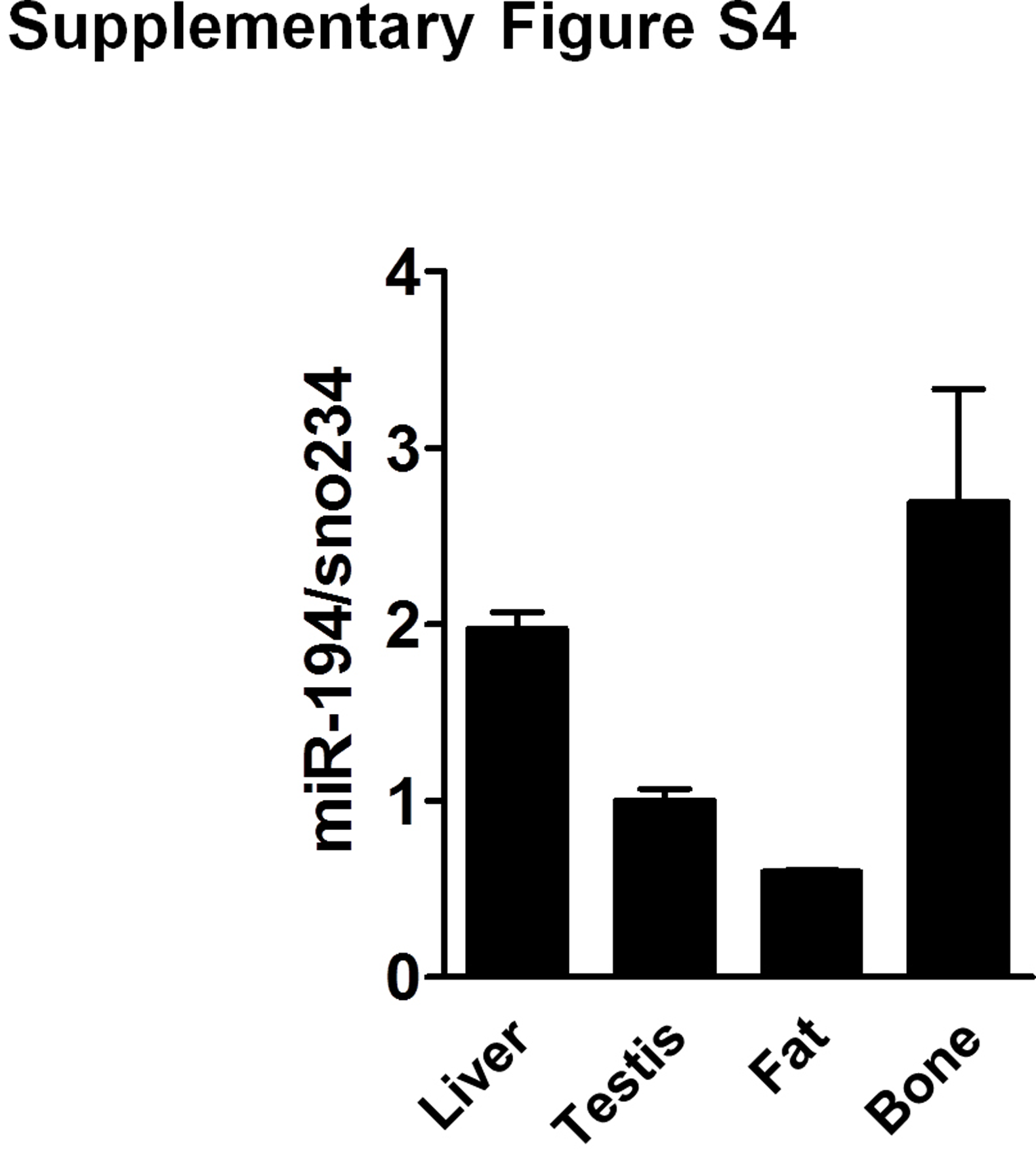

Supplement: Supplementary Figure S4 [file cddis2014485x5.tif]

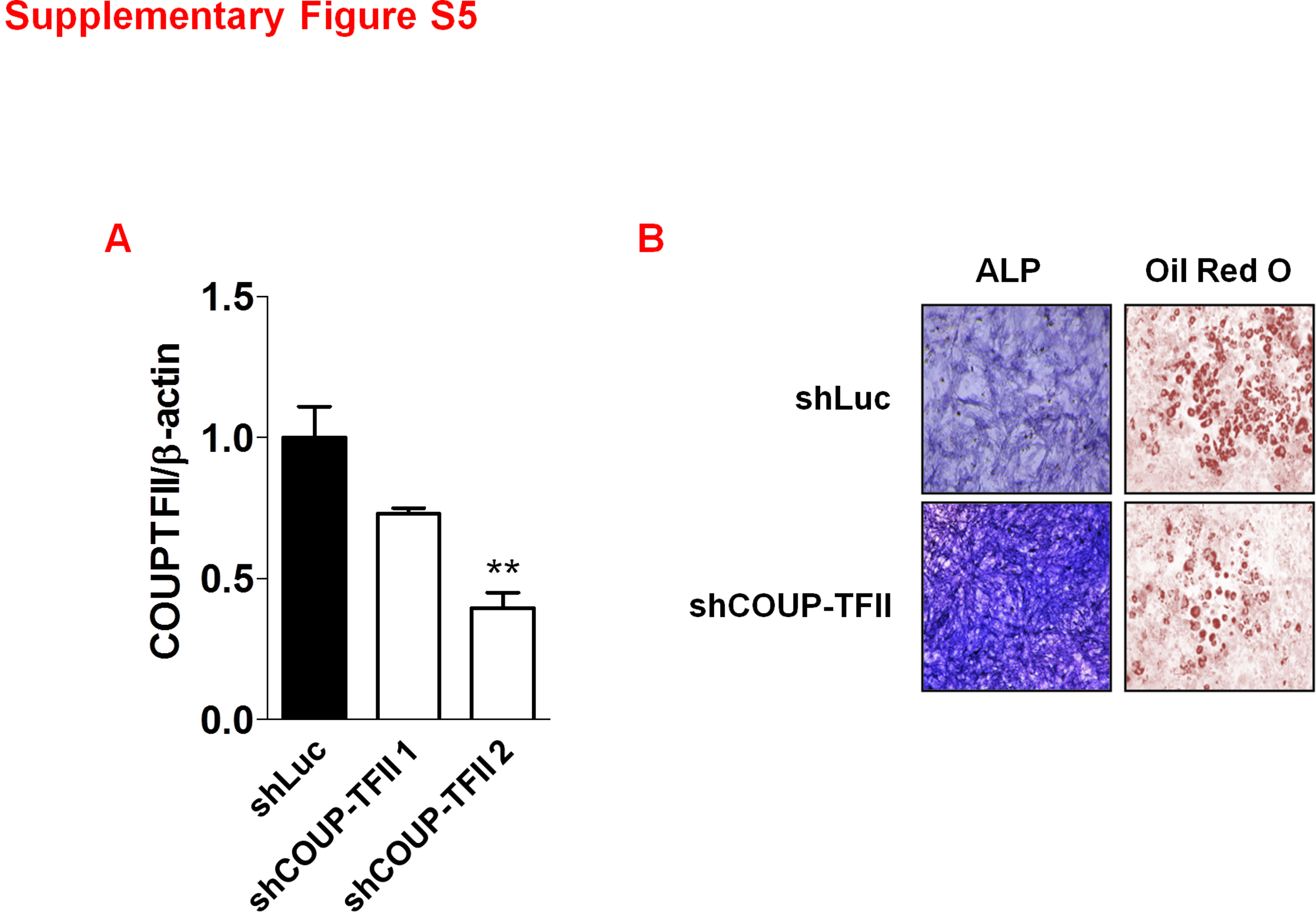

Supplement: Supplementary Figure S5 [file cddis2014485x6.tif]

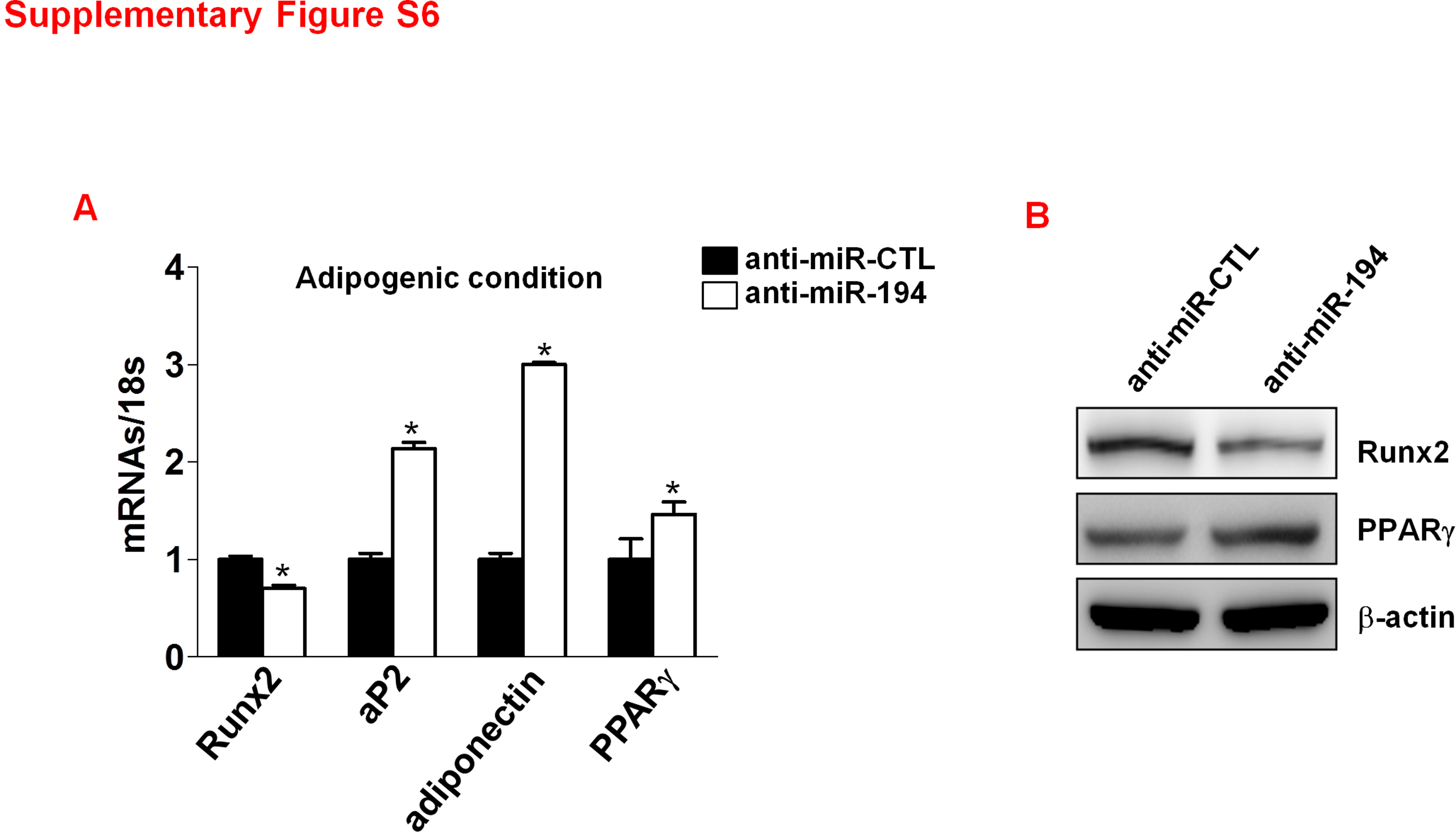

Supplement: Supplementary Figure S6 [file cddis2014485x7.tif]
