# Supplementary material for: MicroRNA-194 reciprocally stimulates osteogenesis and inhibits adipogenesis via regulating COUP-TFII expression
Source: Cell Death Dis. 2014 Nov 20;5(11):e1532–. doi: 10.1038/cddis.2014.485 (PMC4260743; doi:10.1038/cddis.2014.485)
Supplement: Supplementary Tables [file cddis2014485x8.docx]

**Supplementary Tables**

**Supplementary Table 1.** The nucleotide sequences used for cloning

| **cloning or microRNAs** | **Nucleotide sequence** |
| --- | --- |
| mir-194-NR2F2-F (Sac I) | 5′-aaagagctcataaatcaatcaaaataagggggag-3′ |
| mir-194-NR2F2- 604-R (Xho I) | 5′-TTTCTCGAGGAGTTGCCTTTTAAAGTTTTATGTTTTC-3′ |
| miR-194-NR2F2-MT-F | 5′- aaggaacaaacaagtttgct -3′ |
| miR-194-NR2F2-MT-R | 5′- agcaaacttgtttgttcctt -3′ |

**Supplementary Table 2.** The nucleotide sequences used for PCR and qRT-PCR

| **Gene name** |  | **Nucleotide sequence** |
| --- | --- | --- |
| COUP-TFII | forward | 5′-GACTCCGCCGAGTATAGCTG-3′ |
|  | reverse | 5′-GCCCAACACAGGAGTTGTTT-3′ |
| Runx2 | forward | 5′-GAGGGCACAAGTTCTATCTG-3′ |
|  | reverse | 5′-CGCTCCGGCCCACAAATCTC-3′ |
| Osteocalcin | forward | 5′-CTCCTGAGAGTCTGACAAAGCCTT-3′ |
|  | reverse | 5′-GCTGTGACATCCATTACTTGC-3′ |
| β-actin | forward | 5′-TTCTTTGCAGCTCCTTCGTTGCCG-3′ |
|  | reverse | 5′-tggatggctacgtacatggctggg -3′ |
| β-actin  (For real-time PCR) | forward | 5′-ACCCACACTGTGCCCATCTAC-3′ |
|  | reverse | 5′-GCCATCTCCTGCTCGAAGTC -3′ |
| COUP-TFII  (For real-time PCR) | forward | 5′-CAAGGCCATAGTCCTGTTCACC-3′ |
|  | reverse | 5′-CGTACTCTTCCAAAGCACACTGG-3′ |
| PPARγ | forward | 5′-CTGGCCTCCCTGATGAATAA-3′ |
|  | reverse | 5′-GGCGGTCTCCACTGAGAATA-3′ |
| aP2 | forward | 5′-TACATGAAAGAAGTGGGAGTG-3′ |
|  | reverse | 5′-GGTGATTTCATCGAATTCCAC-3′ |

**Supplementary Table 3.** The nucleotide sequences for miRNAs

| **miRNAs** | **Nucleotide sequence** |
| --- | --- |
| mmu-let-7d | 5′-AGAGGTAGTAGGTTGCATAGTT -3′ |
| mmu-miR-17 | 5′-CAAAGTGCTTACAGTGCAGGTAG -3′ |
| mmu-miR-24 | 5′-TGGCTCAGTTCAGCAGGAACAG -3′ |
| mmu-miR-194 | 5′-gctgtaacagcaactccatgtgg -3′ |
| mmu-miR-195 | 5′-TAGCAGCACAGAAATATTGGC-3′ |
| mmu-miR-298 | 5′-GGCAGAGGAGGGCTGTTCTTCCC -3′ |
| mmu-miR-374 | 5′-ATATAATACAACCTGCTAAGTG-3′ |
| mmu-miR-721 | 5′-CAGTGCAATTAAAAGGGGGAA -3′ |
| sno234 | 5′- gcgcggaactgaatctaagtgatttaacaa -3′ |
